# Association of novel intra‐cesarean post‐placental intrauterine device insertion techniques, and early ultrasound, with expulsion rates and strings visibility: A prospective exploratory study

**DOI:** 10.1002/ijgo.71007

**Published:** 2026-04-07

**Authors:** Kyungu M. Kime, Liza Bazzett‐Matabele, G. Justus Hofmeyr

**Affiliations:** ^1^ Department of Obstetrics and Gynecology University of Botswana and Princess Marina Hospital Gaborone Botswana; ^2^ Department of Obstetrics and Gynecology University of Arkansas for Medical Sciences Little Rock Arkansas USA; ^3^ Effective Care Research Unit Walter Sisulu University and University of the Witwatersrand East London South Africa

**Keywords:** expulsion rate, intra‐cesarean post‐placental intrauterine device, long‐acting reversible contraception, predischarge IUD placement, reinsertion, strings visibility, ultrasound evaluation

## Abstract

**Objective:**

To assess an innovative technique of intra‐cesarean post‐placental insertion of the Copper T380A IUD (CPPIUD), and explore the role of predischarge ultrasound with replacement of low‐lying IUDs.

**Methods:**

We conducted a prospective observational study at Princess Marina Hospital, Gaborone, Botswana, from May 31, 2023, to April 3, 2024. Sixty‐seven participants were recruited following CPPIUD. We compared the standard FIGO technique with a novel method in which the insertion straw was left in place to stabilize the IUD and strings during uterine and abdominal closure, then retrieved transvaginally (see youtube video https://www.youtube.com/watch?v=ToWkp3z0_Vg). Technique selection was according to the attending surgeons' personal preference. IUD placement was assessed by predischarge transabdominal ultrasound, and participants were followed up at 6 weeks or later. The primary outcome was IUD retention.

**Results:**

Overall IUD retention was 63/67 (94.0%). The straw‐stabilized group showed a slightly but not statistically significantly higher retention rate (97.9% vs. 85.0%) and string visibility (34.0% vs. 15.0%). Predischarge ultrasound identified low‐lying IUDs in 7/67 participants (10.4%) across both groups; these devices were replaced. One spontaneous expulsion and three removals on request occurred after discharge.

**Conclusion:**

The straw‐stabilized method is at least equivalent to the standard approach. Early predischarge ultrasound may enhance retention.

## 1 INTRODUCTION

FIGO (the International Federation of Obstetrics & Gynecology) promotes postpartum intrauterine device (PPIUD) use, with supportive programs in several countries.^1^ Uptake in sub‐Saharan Africa has been very low (<1% in Tanzania).^1^ At Princess Marina Hospital, most parturients choose condoms, pills, injectable contraceptives, or prefer to choose a method at 6 weeks postpartum.^2^

The innovative Botswana Assistance to Curb Unintended Pregnancies (BACKUP) quality improvement project at Princess Marina Hospital showed a 3.7‐fold increase in IUD use following pregnancy loss or vaginal or cesarean birth.^3^

PPIUD has higher expulsion rates but higher use rates at 12 months when compared with interval insertions.^4^, ^5^

In the absence of manufacturer instructions on the optimal method of “off label” PPIUD placement at cesarean delivery (CPPIUD), practitioners have empirically adopted various methods. Placement technique impacts visibility of the strings and retention of the IUD at follow up.^6^ Non visible strings make removal more difficult.^7^

CPPIUD insertion has expulsion rates of 5%–10% during the first year^8^ and lower expulsion rates may be achieved with training.^6^ Studies in Africa have found an average expulsion rate of 10%.^6^

Reported insertion techniques include manual and sponge forceps,^6^ and a purpose‐designed postpartum insertion tube.^9^ Apart from the hang up technique,^10^ the association of insertion techniques at cesarean with short‐term expulsion has not been fully explored, representing a clinically relevant knowledge gap.

Techniques to increase the likelihood of postpartum string visibility include extending the IUD threads using chromic sutures, partially dividing one string and attaching it to the other to create additional length,^11^ and/or manipulation of strings toward the cervix.^6^, ^11^ Longer strings (such as those of the Cu375 IUD) have a higher probability of being visible at follow up.^12^ PPIUD placement appears to be associated with more positional abnormalities (15%) compared with interval placement. Malpositioned IUDs are best evaluated with the use of ultrasound, preferably three‐dimensional, and hysteroscopy.^13^

A low‐lying IUD after cesarean birth, defined as a fundal gap exceeding 2–3 cm on ultrasound assessment, increases the risk of contraceptive failure, expulsion, and abnormal bleeding. Timely replacement is warranted.^13^

The objectives of the present study were to compare the IUD position, expulsion up to 6 or more weeks, and string visibility following different insertion techniques chosen by surgeons according to their personal preference, and to evaluate immediate replacement of low‐lying IUDs postoperatively. The hypotheses were that a novel method of placement using the IUD straw for stabilization would have more favorable outcomes than the standard manual insertion recommended by FIGO, and that postoperative replacement of low‐lying IUDs would pre‐empt subsequent expulsion.

## 2 MATERIALS AND METHODS

This was a prospective, non‐randomized observational pilot study comparing outcomes between participants with IUD insertion by means of a novel straw‐stabilized method and routine placement. Participants were identified and enrolled after cesarean delivery (usually day 2). Data relating to the cesarean birth were collected from the hospital notes as per the approved study protocol. The study was conducted at Princess Marina Hospital, Gaborone, the main state referral hospital for southern Botswana, among participants who had received CPPIUD placement during the study period and consented to participate in the follow‐up study. This setting was chosen because of large patient numbers and previous research to promote post‐pregnancy IUD use. Recruitment and follow up occurred between May 31, 2023, and April 3, 2024.

The insertion technique was chosen by each surgeon according their usual practice, without any pre‐defined criteria. The standard IUD insertion technique involved manipulation of the IUD strings toward the cervix by the surgeon after placement of the IUD at the fundus either digitally, using a swab‐holding (sponge) forceps or using its inserter followed by immediate removal of the insertor. The three methods were consolidated as they achieved the same fundamental goal: placement of the IUD at the fundus and direction of the loose strings toward the cervix. For the straw stabilization technique, the introducer tube with collar and plunger removed was used to place the arms‐free IUD at the fundus and the lower end was passed through the cervix. The tube stabilized the IUD during the procedure and protected the thread from ensnarement during uterine closure. The tube was retrieved digitally by vaginal examination after completion of the surgery. No additional steps to promote IUD retention, such as suturing it to the fundus, were undertaken. The fundamental difference from previous methods was that the insertion straw remained in the uterus till the end of the operation to stabilize the IUD in the fundal position, protect the strings from ensnarement during uterine closure, and maintain the alignment of the strings toward the cervix. All insertions were performed by obstetrics and gynecology residents in training, medical officers working exclusively in obstetrics and gynecology, and specialist obstetrician gynecologists.

Outcomes were compared between the standard method and the straw stabilization method. Outcomes were recorded as follows. Postpartum, recorded outcomes were (1) IUD expelled from the uterus; (2) ultrasound findings: IUD crossbar more than 10 mm from uterine fundus; IUD past internal cervical os; IUD in lower uterine segment; (3) IUD removed and re‐inserted; and (4) strings visible (only participants having removal). At follow up at 6 or more weeks, the recorded outcomes were the following: (1) history: IUD expelled or removed; (2) ultrasound assessment: IUD in place (primary outcome); IUD past internal cervical os; empty uterus; IUD crossbar more than 5 mm from uterine fundus; and (3) clinical: strings visible with vaginal speculum examination; strings trimmed.

### 2.1 Study procedures

We sought informed consent for participation in the study from patients who had received CPPIUD, usually on the second day after cesarean birth. Patients under 18 years of age or unable to consent were excluded. We approached all patients over 18 years with CPPIUD insertion during the study period, irrespective of uterine anomalies or intraoperative/postoperative complications.

Demographics and clinical variables were recorded and a transabdominal ultrasound for the IUD's location was performed. Speculum examination was only carried out when the IUD was seen toward the external os. Participants were assigned a follow‐up appointment date and received a reminder call and text 1 week before the scheduled visit.

The follow‐up appointment was conducted between 6 and 8 weeks postpartum. Clinical variables related to IUD placement were assessed, followed by a vaginal speculum examination and transvaginal, or if unsatisfactory, transabdominal ultrasound.

As outlined in the consent form, participants could contact the first author directly regarding any IUD‐related symptoms and could arrange an earlier review if necessary.

Ultrasound examinations were performed by the first author. For the transabdominal ultrasound, the location of the IUD on sagittal and transverse cross‐section and, when possible, coronal views was sought. The distance from the fundus to the IUD's crossbar, axial orientation, and distance from the tip of the vertical arm to the internal cervical os were recorded.

Immediate postpartum clinical data included level of pain, amount of bleeding or lochia, noted expulsion of the IUD, noted strings, perforation, and infection occurrence. The follow‐up appointment clinical data included duration and amount of lochia, report of spotting or bleeding, ability to palpate strings, pain, duration and heaviness of menstruation, and noted expulsion.

Speculum examination included strings visibility, length, and if not visualized retrieval with a throat swab or cytobrush. Trimming of strings was done when more than 2 cm projection from the cervix was noted.

For the vaginal ultrasound scan, sagittal, coronal, and transverse views were obtained.

### 2.2 Sample size

This was a pilot study using convenience prospective sampling because the outcomes according to method of insertion were not documented previously and we were unable to anticipate how many participants would undergo each insertion technique. To our knowledge, no previous randomized or observational studies have compared these different methods.

This pilot study aimed to provide outcome estimates to determine the sample size needed for a definitive trial, and whether such a definitive trial would be feasible and justified.

### 2.3 Data collection

The obstetrics register was screened for an indication that an IUD had been placed at cesarean birth. Theater notes were reviewed and clarification was sought from the surgeon if necessary. Physical and ultrasound examinations were performed before discharge and at the postnatal visit. Phone calls and text messages were used to establish rapport with participants. This approach enabled the first author to receive relevant information from participants who were unable to attend their scheduled appointments but who had follow up at local clinics. Ultrasound and physical examination reports from the nearest facility were received via email, WhatsApp, or Snap Photo. Data obtained included ultrasound reports and reports of IUD‐related symptoms, string visibility, or IUD removal.

### 2.4 Ethical considerations

The protocol was approved by the University of Botswana Institutional Review Board (reference UBR/RES/IRB/BOI/GRAD/326) on February 10, 2023, and the Princess Marina Ethics and Research Committee (reference PMH 2/11AII [174]) on May 25, 2023, before any trial‐related procedures were performed. Comprehensive informed consent forms were given to participants, which explained the potential risks, discomforts, and benefits associated with the study, the purposes of the study, and that participation was voluntary and could be withdrawn without giving reasons and with no loss of benefits.

The data were kept confidential. The electronic data were stored on a password‐protected laptop. The case report forms and signed consent forms were kept in a locked cupboard. This study report is presented without identifying information of participants. The study procedures were piloted with three participants, not included in the final analysis, to ensure the feasibility of the procedures.

### 2.5 Data processing and analysis

Data were entered onto an Excel spreadsheet, checked for accuracy, and analyzed using EpiInfo software. Baseline and outcome information was summarized in tables, with variables presented as means with standard deviations, or proportions with percentages and 95% confidence intervals. Categorical variables were compared using the χ^2^ test or, for comparisons involving small numbers, Fisher exact test. Statistical significance was presented as *P*‐values, and risk ratios with 95% confidence intervals were reported to quantify effect sizes. Given the limited sample size of this exploratory study, adjustments for potential confounders or missing data were not considered useful.

## 3 RESULTS

Baseline characteristics were generally comparable between the two groups with respect to age, macrosomia, breech presentation, prolonged labor, or complications such as pre‐eclampsia or antepartum hemorrhage, except that a higher proportion of participants in the straw‐stabilized group had repeat cesarean births (87.2% versus 60.0%, *P* = 0.03) (Table 1).

**TABLE 1:** Baseline data.^a^

Characteristic	Insertion technique				*P* value
	Straw via vagina (*n* = 47)		Standard insertion (*n* = 20)		
		95% CI		95% CI	
Age, years	30.7 ± 5.8		30.8 ± 5.9		0.24
Multiparous	42 (89.4%)	76.7%–95.9%	15 (75.0%)	53.3%–88.8%	0.26
Elective cesarean	29 (61.7%)	47.4%–74.2%	12 (60.0%)	38.7%–78.1%	1.00
Labor cesarean	18 (38.3%)	25.8%–52.6%	8 (40.0%)	21.9%–61.3%	1.00
Multiple pregnancy	1 (2.1%)	0.4%–11.1%	0 (0.0%)	0.0%–16.1%	1.00
Macrosomia (>4000 g)	2 (4.3%)	1.2%–14.2%	2 (10.0%)	2.8%–30.1%	0.58
Mean birth weight, g	3193 ± 654		3212 ± 465		0.87
Repeat cesarean	41 (87.2%)	74.8%–94.0%	12 (60.0%)	38.7%–78.1%	0.03
Prolonged labor	3 (6.4%)	2.2%–17.2%	4 (20.0%)	8.1%–41.6%	0.18
Breech presentation	2 (4.3%)	1.2%–14.2%	2 (10.0%)	2.8%–30.1%	0.58
Failed induction	1 (2.1%)	0.4%–11.1%	0 (0.0%)	0.0%–16.1%	1.00
Fetal distress	3 (6.4%)	2.2%–17.2%	1 (5.0%)	0.9%–23.6%	1.00
Pre‐eclampsia with severe features	1 (2.1%)	0.4%–11.1%	2 (10.0%)	2.8%–30.1%	0.21
Fresh scar	1 (2.1%)	0.4%–11.1%	0 (0.0%)	0.0%–16.1%	1.00
Antepartum hemorrhage	1 (2.1%)	0.4%–11.1%	0 (0.0%)	0.0%–16.1%	1.00

^a^ Data are expressed as absolute counts (%) with 95% confidence intervals, or as mean values ± standard deviation. *P*‐values are given for comparisons.

### 3.1 Immediate postoperative outcomes

Overall expulsion or malposition rates were 9/67 (13.4%), with no significant difference between straw stabilization (5/47, 10.6%) and standard technique (4/20, 20.0%) (Table 2). Of these, one in each group was expelled in theater with blood clot from an atonic uterus and replaced in theater: straw stabilization 1/47 (2.1%) and standard technique 1/20 (5.0%). For the remaining seven, on postoperative ultrasound the IUD was seen in the lower uterine segment or below the internal cervical os, with no significant difference between the straw stabilization (4/47, 8.5%) and standard insertion technique (3/20, 15.0%) groups. All seven participants agreed to replacement of the IUD. Among those requiring replacement, strings were visible for 4/4 (100%) in the straw stabilization group and 2/3 (66.7%) in the standard technique group.

**TABLE 2:** Postoperative assessment before hospital discharge by insertion technique.^a^

Events	Insertion technique		Comparisons		
	Straw via vagina	Standard			
	(*n* = 47)	(*n* = 20)	RR	95% CI	*P* value
Expelled and re‐inserted in theater	1 (2.1%)	1 (5.0%)	0.43	0.03–6.5	0.51
Ultrasound assessment					
Crossbar >10 mm from fundus	20 (43.0%)	7 (35.0%)	1.2	0.61–2.4	0.60
Malposition^b^ IUD in lower segment	1 (2.1%)	1 (5.0%)	0.43	0.03–6.5	0.51
Malposition^b^ IUD past internal os	3 (6.4%)	2 (10.0%)	0.64	0.12–3.5	0.63
Clinical					
IUD removed and re‐inserted	4 (8.5%)	3 (15.0%)	0.57	0.14–2.3	0.42
Strings visible for removal	4/4 (100%)	2/3 (66.7%)	1.5	0.67–3.3	0.43

Abbreviations: CI, confidence interval; IUD, intrauterine device; RR, risk ratio.

^a^ Outcomes expressed as proportions (%) and comparisons expressed as risk ratios with 95% confidence intervals and probability (*P*).

^b^ Malposition: IUD ≥30 mm from fundus.

All nine participants with re‐insertion had the IUD in place at the interval visit.

All 67 participants attended postpartum follow‐up visits at 6 or more weeks at either a primary clinic or the hospital (Table 3).

**TABLE 3:** Follow up after 6 weeks.^a^

	Insertion technique		Comparisons			Totals
	Straw via vagina	Standard				
			RR	95% CI	*P*	Count (%)
Assessment and ultrasound by first author	(*n* = 23)	(*n* = 12)				(*n* = 35)
IUD in place	23 (100%)	10 (83.3%)	1.2	0.93–1.5	0.11	33 (94.3%)
IUD past internal os removed and re‐inserted	0 (0%)	1 (8.3%)	0	Undefined	0.34	1 (2.9%)
Empty uterus	0 (0%)	1 (8.3%)	0	Undefined	0.34	1 (2.9%)
Crossbar to fundus ≥5 mm	1 (4.3%)	0 (0%)	Undefined	Undefined	1.00	1 (2.9%)
Visible strings speculum	11 (47.8%)	2 (16.7%)	2.9	0.76–11	0.14	13 (37.1%)
Strings trimmed	7 (30.4%)	0 (0%)	Undefined	Undefined	1.00	7 (20.0%)
Telephonic interview by first author plus information from clinic	(*n* = 24)	(*n* = 8)				(*n* = 32)
IUD removed at clinic at 8 d for vaginal bleeding	1 (4.2%)^b^	1 (12.5%)^c^	0.33	0.024–4.7	0.44	2 (6.3%)
Reported IUD in place	23 (95.8%)	7 (87.5%)	1.1	0.83–1.4	0.44	30 (93.7%)
Reported strings visualized at clinic	5 (21%)	1 (12.5%)	1.7	0.23–12	1.00	6 (18.8%)
Removed at clinic at 6 week	1 (4.2%)^d^	1 (12.53%)	0.33	0.02–4.7	0.44	2 (6.3%)
Combined in person and telephonic assessment	(*n* = 47)	(*n* = 20)				(*n* = 67)
IUD in place	46 (97.9%)	17 (85.0%)	1.15	0.95–1.4	0.08	63 (94.0%)
Strings visible	16 (34.0%)	3 (15.0%)	2.3	0.74–6.9	0.15	19 (28.4%)

Abbreviations: CI, confidence interval; IUD, intrauterine device; RR, risk ratio.

^a^ Outcomes expressed as proportions (%) and comparisons expressed as risk ratios (RR) with 95% confidence intervals (CI) and probability (*P*).

^b^ Intrauterine position confirmed with ultrasound, strings visible 4 cm. Contraception undecided.

^c^ Strings visible for removal. Contraception injectable progestogen.

^d^ Strings uncomfortable down to introitus. Apparently not offered string trimming.

### 3.2 Straw stabilization technique

Of the 47 participants, 23 (48.9%) were seen for postpartum follow up at Princess Marina Hospital and all had the IUD in place. The mean crossbar to fundus measurement was 3 ± 1.2 mm. Strings were visible in 11/23 (47.8%) and trimmed in 7/23 (30.4%).

Of 47 participants, 24 (51.1%) were reviewed at their local clinics. Of these 24, 23 (95.8%) had the IUD in place and 5 (20.8%) reported strings being visible. Two of 24 (8.3%) had their IUD removed at local clinics. One underwent removal on day 8 post‐cesarean due to bothersome bleeding; ultrasound confirmed the IUD in place and the strings were reported as visible. The participant was considering bilateral tubal ligation. The second IUD was removed at the 6‐week follow up due to discomfort attributed to untrimmed strings, and the participant elected to initiate etonogestrel implant use.

Based on the combined review at the hospital and reported clinic review, at 6 or more weeks 46/47 (97.9%) IUDs were still in place. Following one removal on request for worrisome strings this reduced to 45/47 (95.7%) and strings were visible in 16/47 (34.0%).

All 5 (10.6%) participants who had the IUD re‐inserted in the perioperative period had the IUD in place at interval check‐up. Strings were visible in three (60.0%) and did not require trimming.

### 3.3 Standard insertion technique

Of the 20 participants, 12 (60.0%) were seen for postpartum follow up at Princess Marina Hospital and 10 (83.3%) had the IUD in place. The mean crossbar to fundus measurement was 3.5 mm, and strings were visible in 2/12 (16.7%) and not trimmed. One (8.3%) woman likely had an expulsion and requested etonorgestrel implant contraception. Transabdominal as well as transvaginal ultrasound could not locate the IUD. Abdominal migration appeared unlikely. Her predischarge ultrasound measurement was 8.1 mm from the fundus to crossbar and 35 mm to internal cervical os. The second participant had a low‐lying IUD with the IUD tip having passed the internal os for 6.2 mm. Strings were visible at the level of vagina introitus and estimated to be 7 cm long. The participant requested reinsertion of a copper T380A IUD.

Of 20 participants, 8 (40.0%) were reviewed at their local clinics. Of these eight, seven (87.5%) were reported to have the IUD in place and no strings were seen. One had had the IUD removed on day 8 for bleeding‐related symptoms. She had chosen depot medroxyprogesterone acetate for contraception.

Altogether, 18/20 (90.0%) were still using the copper T IUD with 3/20 (15.0%) having visible strings at interval check‐up.

The four participants in the standard technique group who had the IUD reinserted following expulsion or malposition in the perioperative period were still using the IUD at the interval visit, of whom one (25.0%) had visible strings.

### 3.4 Comparisons

There were no statistically significant differences between the straw stabilization group and the standard insertion technique for IUD retention (97.9% vs. 85%) and string visibility (34.0% vs. 15.0%).

## 4 DISCUSSION

This prospective observational study showed that the novel method of stabilizing the IUD and strings with the IUD straw during cesarean birth may be at least equivalent to the standard approach, and further adequately powered randomized studies are needed to determine superiority. Differences in expulsion and string visibility rates need to be interpreted with caution as they were not statistically significant. Future studies might address whether IUD expulsion in theater with expulsion of clots from an atonic uterus might be avoided by first expelling clots and “rubbing up” the uterus before removing the stabilizing straw. The study also raises the possibility that day 2 ultrasound examination is useful to identify and replace low‐lying IUDs. This proactive management might have contributed to the low rate of later expulsions.^14^, ^15^ Overall, IUD continuation was high (94%). Only one spontaneous expulsion occurred post‐discharge. However, routine ultrasound has resource implications and may not be generalizable to all settings. The higher rate of repeat cesarean in the straw‐stabilized group (87.2%) compared with the standard method group (60%) may have influenced outcomes, though we are not aware of evidence of an effect of previous cesarean in either direction. Expulsion rates were favorable compared with previous reported rates of 5%–10%^2^ and 10%^6^ in Africa.

As participants were enrolled in the study postoperatively and the IUD insertion techniques were not randomly allocated, the study was subject to selection bias. The choice of IUD insertion method was according to the personal preferred method of the surgeon, not the intraoperative findings. The study's limitations included limited sample size (so it was not powered to detect clinically relevant differences), lack of randomization, lack of standardization of IUD insertion techniques used by multiple operators, potential for observer bias affecting subjective outcomes due to lack of blinding of outcome assessments, inability to assess inter‐observer reliability for observations at peripheral clinics, and reliance on telephonic follow up for nearly half of the participants for the interval check‐up, validated by reports received from the clinics attended by the participants. As the IUD insertion procedures were not part of the research intervention, there was no ethical approval to standardize techniques.

In conclusion, the present study highlights the feasibility of intra‐cesarean post‐placental IUD insertion. Postoperative ultrasound assessment offered the opportunity to remedy malposition in the immediate postpartum period within the safety time frame of 48 h after birth. The use of the IUD straw to stabilize the IUD and strings during completion of the surgery represents a potentially useful innovation.

The procedure may be viewed on YouTube Obstetric Skills library: https://www.youtube.com/channel/UCrbUWWB7XW7fpZ6YzISmOuw/about, https://www.youtube.com/watch?v=ToWkp3z0_Vg “Intrauterine contraceptive device insertion at Caesarean section”. The IUD insertion method is demonstrated at timecodes 3:56 to 4:06, and from 9:15 to the end.

## AUTHOR CONTRIBUTIONS

KK wrote the first draft of the protocol and paper and conducted the clinical study. LB and GJH supervised the clinical study, data analysis, and manuscript writing. GJH conceived the straw stabilization method of IUD insertion. All authors approved the final version of the manuscript.

## FUNDING INFORMATION

None.

## CONFLICT OF INTEREST STATEMENT

GJH previously received payments as inventor of the Maternal Well tray for blood loss monitoring after birth but has no current or future financial interest in the device. The authors have no other conflicts of interest.

## Data Availability

The data will be available from the first author upon reasonable request after publication of the primary paper.
